# Mapping Brucellosis Increases Relative to Elk Density Using Hierarchical Bayesian Models

**DOI:** 10.1371/journal.pone.0010322

**Published:** 2010-04-23

**Authors:** Paul C. Cross, Dennis M. Heisey, Brandon M. Scurlock, William H. Edwards, Michael R. Ebinger, Angela Brennan

**Affiliations:** 1 Northern Rocky Mountain Science Center, United States Geological Survey, Bozeman, Montana, United States of America; 2 National Wildlife Health Center, United States Geological Survey, Madison, Wisconsin, United States of America; 3 Wyoming Game and Fish Department, Pinedale, Wyoming, United States of America; 4 Wyoming Game and Fish Department, Laramie, Wyoming, United States of America; 5 Big Sky Institute, Montana State University, Bozeman, Montana, United States of America; 6 Department of Ecology, Montana State University, Bozeman, Montana, United States of America; University of Utah, United States of America

## Abstract

The relationship between host density and parasite transmission is central to the effectiveness of many disease management strategies. Few studies, however, have empirically estimated this relationship particularly in large mammals. We applied hierarchical Bayesian methods to a 19-year dataset of over 6400 brucellosis tests of adult female elk (*Cervus elaphus*) in northwestern Wyoming. Management captures that occurred from January to March were over two times more likely to be seropositive than hunted elk that were killed in September to December, while accounting for site and year effects. Areas with supplemental feeding grounds for elk had higher seroprevalence in 1991 than other regions, but by 2009 many areas distant from the feeding grounds were of comparable seroprevalence. The increases in brucellosis seroprevalence were correlated with elk densities at the elk management unit, or hunt area, scale (mean 2070 km^2^; range  = [95–10237]). The data, however, could not differentiate among linear and non-linear effects of host density. Therefore, control efforts that focus on reducing elk densities at a broad spatial scale were only weakly supported. Additional research on how a few, large groups within a region may be driving disease dynamics is needed for more targeted and effective management interventions. Brucellosis appears to be expanding its range into new regions and elk populations, which is likely to further complicate the United States brucellosis eradication program. This study is an example of how the dynamics of host populations can affect their ability to serve as disease reservoirs.

## Introduction

The relationship between host density and parasite transmission is fundamental to understanding infectious disease dynamics as well as implementing control strategies [1], [2]. Models predict that when transmission is directly proportional to host density the parasite will be unable to persist when the host density is reduced below some threshold [1], [3], [4]. This forms the basis for using public health practices such as social distancing (e.g., school closures) to reduce the spread of pandemics [5]. In wildlife systems, this density-transmission relationship is the justification for strategies that aim to reduce the density of susceptible individuals (e.g., culling, increasing hunter quotas, sterilization and vaccination) [6], [7]. However, empirical evidence for host population thresholds remains limited, and few studies have directly evaluated the functional relationship between contact rates and host density [6], [8], [9]. In many social species we expect contact rates, and thus transmission rates, to saturate as group sizes may remain relatively constant while population sizes increase [10]. If so, managers may need to reduce host densities to low levels before those reductions have an impact upon disease dynamics.

One reason for the lack of empirical evidence in natural systems is the inherent difficulty of matching disease data with variation in host density in either space or time. This is particularly challenging in chronic diseases of wildlife species because of the logistics associated with collecting data at large geographic scales over long time periods. Therefore, most datasets are either long-term studies of focal populations or broad-scale studies of more limited duration making temporal patterns difficult to detect. In this study, we use a 19-year dataset of brucellosis in Wyoming elk to investigate the relationship between host density and disease dynamics. In particular, we assess how spatial variation in elk density correlates with spatial differences in brucellosis increases over time (i.e., a space by time interaction).

**Figure 1:**
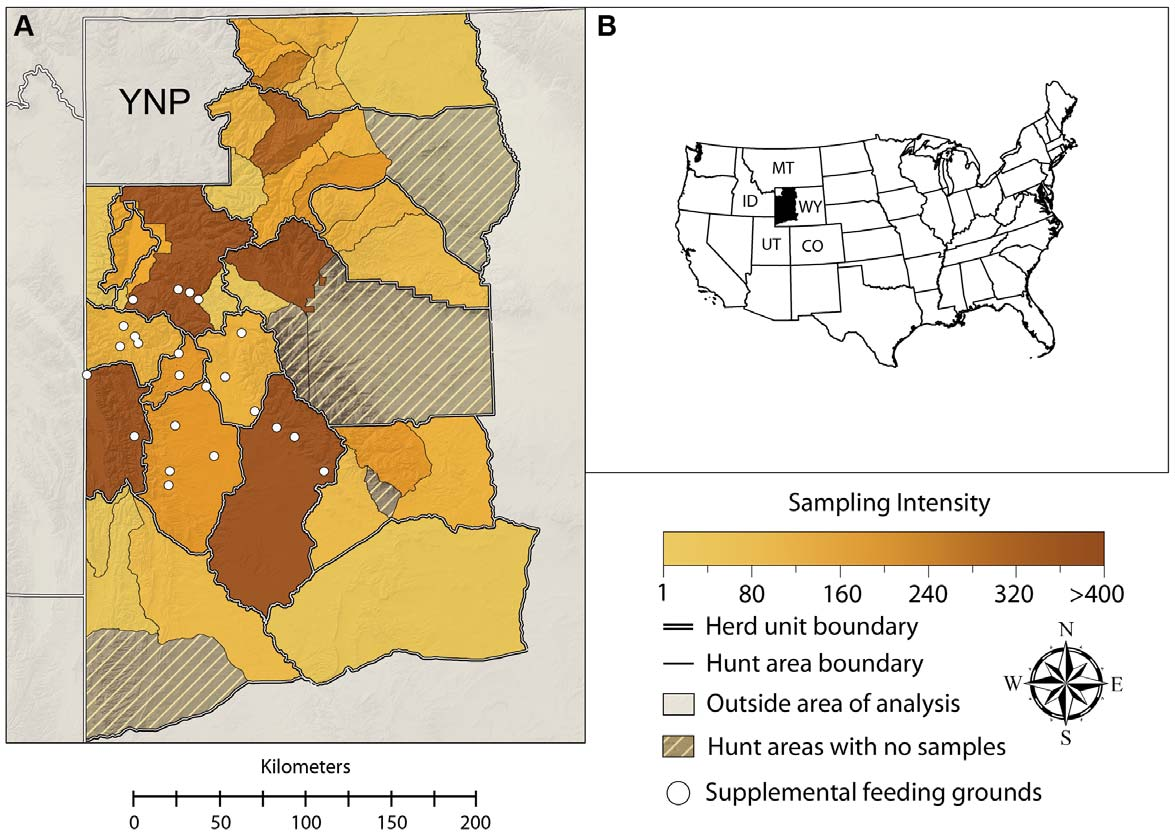
Map of the study area. In (A), the shading indicates the intensity of brucellosis testing among adult female elk in each hunt area. Broad and fine scale spatial analysis units (herd units and hunt areas, respectively) are shown along with the location of the 23 supplemental feeding grounds. Hashed regions did not have any disease test results. (B) The location of the study area within the United States.

Brucellosis, a bacterial disease caused by *Brucella abortus*, is a major wildlife/livestock issue in the Greater Yellowstone Ecosystem (GYE) [11] and in many countries worldwide where it also remains a human public health problem [12]. Disease management in the GYE is complex, involving several state and federal agencies, and multiple mitigation strategies. For example, roughly 35% of the Yellowstone bison population was lethally removed in 2008 to limit the potential for disease transmission from bison to cattle as bison attempt to migrate out of the park during winter. Despite this extensive management of bison, cattle herds in Wyoming, Idaho and Montana have been infected since 2004 and the available data suggest that these infections were due to elk [13], [14], primarily due to the limited interactions between bison and cattle. In the southern portions of the GYE, elk are supplementally fed during winter at 23 feeding grounds (Fig. 1). Brucellosis seroprevalence in elk using supplemental feeding grounds in winter varies from 10–35% [15], [16], while unfed elk populations around the GYE historically had brucellosis seroprevalence values of 2–4% [17], and brucellosis was not known to persist in elk populations outside the GYE [18]. The supplemental feeding grounds are intended to prevent the movement of elk onto agricultural land and thus minimize contact between elk and cattle during winter. A by-product of this management activity is increased aggregation of elk between November and April. Until recently, there was a consensus that *B. abortus* is not self-sustaining in unfed elk populations [18], but recent research suggests that some unfed elk populations now maintain brucellosis at a seroprevalence of greater than 10% [16], [19].

*Brucella abortus* causes abortions in female hosts and is transmitted within and among wildlife and livestock when individuals investigate or feed near infected fetuses, placentas or birthing fluids [18]. Fifty to sixty percent of infected female elk abort their first calf post-infection [20], but only one in nine elk lose a second calf [21]. Studies in elk [20], bison [22], and cattle [23], [24], [25] have failed to show sexual transmission of *B. abortus*. In both bison and elk, calves born to infected mothers tend to be initially seropositive but are seronegative by six months old suggesting maternal antibodies rather than vertical transmission [20], [26]. Although *Brucella* spp. can survive in moist, dark environments for up to two months, recent work in Wyoming suggests that in areas with abundant scavengers, fetuses are typically consumed within 24–48 hours [27]. Brucellosis has not been shown to have an effect on survival of elk or bison [18], [28], [29].

Previously, Cross *et al.* [19] showed that elk were maintaining higher levels of brucellosis in new regions of the GYE and assessed several of the potential causes. Those analyses were conducted at a broad herd unit (HU) scale (range  = 770–11220 km^2^) and did not include regions where elk are supplementally fed during the winter, which simplified analyses and increased the number of samples per spatial unit [19]. However, these analyses did not assess the heterogeneity within herd units, compare across regions with and without feeding grounds, or estimate the relationship between seroprevalence and elk density. Here we analyze a more comprehensive dataset at the finer spatial scale of hunt areas (HA) which are nested within herd units (HU), using a hierarchical Bayesian methodology that allows for the correlation among adjacent regions [30]. The Bayesian approach and Markov chain Monte Carlo (MCMC) estimation provides a framework for quantitative predictions of unsampled or weakly sampled regions. This approach has been adopted in several human disease studies [31], [32] and is becoming more common in wildlife disease studies [33], [34].

## Materials and Methods

We used two datasets of elk brucellosis seroprevalence from Wyoming provided by the Wyoming Game and Fish Department (WGFD). The first dataset consisted of elk blood samples collected by hunters from 1991 to 2008 across Wyoming, and the second dataset consisted of elk captured for research and management purposes on supplemental feeding grounds from 1993 to 2009. Elk were classified as calves, yearlings or adults (≥2 yrs old) based on incisor tooth eruption patterns. This dataset also contained 320 tests of individuals that were sampled multiple times, but for simplicity we used only one randomly chosen test per individual. From these two datasets, we subset the data to include only adult female elk within the brucellosis endemic area (Fig. 1), which we defined as areas that had seropositive elk. By focusing on adult females, we reduced confounding due to age and sex while at the same time utilizing the population segment most relevant to transmission of *B. abortus* and cattle risk. Our final dataset included 6458 tests and 744 positive cases.

All samples were assigned to hunt areas, which were nested within larger herd units (Fig. 1). Several supplemental feeding grounds were located near hunt area boundaries and we did not have the data necessary to confidently assign the elk captured on those feeding grounds to hunt areas where they would most likely be located during the hunting season. Therefore, we combined several hunt areas around the feeding grounds into larger spatial units, although we still refer to them as hunt areas in the analysis (Fig. 1, S1, and Table S1). This amalgamation involved some subjectivity, but we caution against over-interpreting minor differences among regions as marked elk have been observed to move across some of the hunt area boundaries (WGFD unpublished data).

The data collection methods have been described elsewhere [15], [16], [19], which we briefly summarize here. Serological assays for both datasets were conducted and interpreted using current National Veterinary Services Laboratories protocols for the card test, plate agglutination, rivanol precipitation–plate agglutination, fluorescence polarization assay using tubes, and complement fixation. A competitive ELISA (cELISA) was used to discriminate vaccine from field strain titers [35]. Reactors were those animals with positive card tests, rivanol ≥1∶25 or higher, CF of 2+ at 1∶20, and SPT ≥1∶100 or higher. Serological profiles were categorized using the United States Department of Agriculture's brucellosis eradication uniform methods and rules for cervids (APHIS 91-45-013). Less than 1% of the serological tests were categorized as suspect, which we included as positive test results. These serological tests indicate whether or not an individual has been exposed, but not whether they are currently infected; and thus serve only as indices of exposure rather than the percentage of individuals that are infectious.

We used elk count data collected at the hunt area scale from WGFD 2004–2007 Job Completion Reports [JCRs, 36]. JCRs summarize annual elk population counts (i.e., trend counts) and counts by age and sex (i.e., classification counts) conducted by WGFD biologists via fixed-wing aircraft, helicopters, or on the ground. We used the most recent population trend count since 2004 divided by the total area (km^2^) of the hunt area to estimate elk density (Table S1, Fig. S1). This is a crude approximation because many hunt areas include unsuitable habitat and the elk counts also include sampling error and change over time. Ideally, one would also account for the temporal variability in elk densities, but relating temporal changes in host density to corresponding changes in disease prevalence is potentially complicated by time lags and we did not believe there was enough temporal variation in seroprevalence to estimate those lags. In addition, more refined data on age are needed to appropriately account for the unknown conversion times [34]. In the discussion we highlight some future research projects that could further refine this analysis. Trend counts were unavailable for four areas around the National Elk Refuge and Grand Teton National Park. However, in these areas the regional biologists considered the classification counts as good surrogates for total elk counts and our conclusions and parameter estimates remained the same whether or not these areas were included in the analysis.

### Statistical analyses

Our response variable was the exposure status *Y_ij_*, determined by serology, for individual elk *i* in site *j*. We assumed that *Y_ij_* was a Bernoulli trial with a probability of being test positive *p_ij_*. We then used a logit link function to relate the probability of infection to covariates. Let *δ_j_* represent the site-specific intercept (log odds). Let *X_ij_* and *Z_ij_* be covariate (row) vectors associated with elk *ij*, and let *t_ij_* be the number of years since 1991 that the sample was taken. Let ***β*** be a (column) vector of regression coefficients (log odds ratios) associated with the time-invariant covariates *X_ij_*, such that time-invariant covariate effects were modeled as *X_ij_* ***β***. Let *φ_j_* be the site-specific time effects, or slopes, (log odds ratios) for year, such that the year effect was modeled as *φ_j_t_ij_*. To allow for time-varying covariate effects, we included a term, *Z_ij_* ***α*** *t_ij_*, where ***α*** is a (column) vector of regression coefficients (log odds ratios) associated with the vector *Z_ij_* of covariates reasonably modeled with time-varying coefficients. Thus, our models were of the general form: 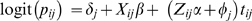.

Our covariates included sampling year (rescaled so that 1991 was the intercept), fed vs. unfed, hunt area (HA) and herd unit (HU), where hunt areas (fine scale) were nested within herd units (broad scale). These covariates could affect either the intercept (i.e., 1991 seroprevalence) or the slope (i.e., time effect; Table 1). Fed areas included at least one supplemental elk feeding ground (Fig. 1). We also suspected that samples that were collected on the feeding grounds between February and April may be more likely to be test-positive than hunter-killed samples that were collected in September to December due to the association between brucellosis and late pregnancy [18]. Therefore we included an indicator variable that denoted whether the sample came from a hunter (Hunt  = 1) or from captures on the feeding grounds (Hunt  = 0). One of the analysis units (HA 97 & 98) included three supplemental feeding grounds where managers have been testing and removing seropositive elk beginning in 2006. We allowed the time effect to differ between 1991–2006 and 2007–2009 for this region by including an additional parameter, *α*_tr_, that was multiplied by an indicator variable that equaled one for this region from 2007–2009 and zero everywhere else.

**Table 1:** Comparison of hierarchical Bayesian logistic regressions of 6458 brucellosis test results of adult female Wyoming elk.

Model	Intercept^1^	Slope	DIC^2^	ΔDIC	pD^3^	Deviance
1	HA, Fed, Hunt	HA, TR, Pop	3897.7	0	34.6	3863.0
2	HA, Fed, Hunt	HA, TR	3899.4	1.7	36.4	3863.0
3	HA, Fed, Hunt	HA, TR, Pop*^θ^*	3899.5	1.9	34.5	3865.0
4	HA, Fed, Hunt	HA, TR,	3899.6	1.9	34.4	3865.2
		*ω* ^1^Pop/(1+*ω*_2_Pop)				
5	HA, Fed,	HA, TR,	3901.3	3.7	34.3	3867.0
	Pop, Hunt	Fed, Pop				
6	HA, Fed,	HA, TR,	3902.1	4.5	33.4	3868.7
	Pop*^θ^*, Hunt	Fed, Pop*^θ^*				
7	HU, Fed, Hunt	HU, Pop, TR	3911.4	14	23.8	3887.7

1 Dependent variables that affected the intercept (i.e., 1991 seroprevalence) and the slope (i.e., time effect). HA  =  Hunt area; HU  =  Herd unit; TR  =  test and dremove, Fed  = 1 for areas with a supplemental feedground, otherwise 0; Pop  =  elk/km^2^: *θ* =  non-linear effect of density.

2 Deviance information criterion

3 pD = 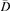-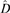, and is an approximation of the model complexity.

Although we restricted our analyses to adult females, we did not have refined age data to account for how shifting age structures may affect overall seroprevalence. However, our previous analyses suggest that even extreme changes in elk age structure are unlikely to shift seroprevalence from 2% to >10%, which is representative of the large changes we detected [19]. We first developed a suite of models at the hunt area scale and then compared the best of those models with equivalent models conducted at the herd unit scale (Fig. 1).

We treated the site-specific terms, *δ_j_* and *φ_j_*, in two ways: either we assumed that all regions are exchangeable with one another given the same covariates [EX, 37], or we used the Besag-York-Mollie spatial convolution approach to account for the spatial correlations among neighboring hunt areas [BYM, 30]. The BYM approach models the spatial effect of region *j* as the sum of a spatially dependent component *δ_sj_* and a spatially independent, or heterogeneity, component *δ_hj_*, *δ_j_* = *δ_sj_*+*δ_hj_*. We assumed that the set neighboring regions for hunt area *j*, {*j*}, were those hunt areas that shared boundaries with area *j*. If *n_j_* is the number of neighboring areas, then 
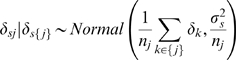
.

We assumed that *δ_hj_* was normally distributed with a mean of *μ* and variance of 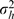. The BYM approach allows one to assess both the extent and total amount of spatial dependence [31] and the relative importance of spatial dependence compared to random heterogeneity can be accessed via the metric 
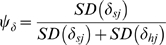
 [38]. We followed a similar approach for the slope parameters, *φ_j_*.

In our hierarchical models, we incorporated host density into the region-specific intercept and slope parameters, *δ_j_* and *φ_j_*, (or *δ_hj_* and *φ_hj_* in the BYM approach). First we assumed a linear effect of density such that 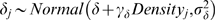 and 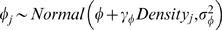, where the unsubscripted terms *δ* and *φ* are omnibus intercepts. Secondly, we incorporated non-linearity by raising elk density to the power *θ*. For values of *θ* between zero and one the effects of elk density increases at a sublinear rate–as density increases, a unit increase in density results in a progressively smaller increase in effect. When *θ* equals one density effects are linear. Finally, we also considered a type II functional response whereby 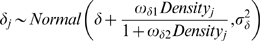 and 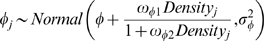. We lacked information on elk density for two hunt areas, however, these areas also lacked any disease testing data (Figs. 1 and S1, Table S1). For these regions we inserted the mean elk density observed across all the other regions.

Where possible we used uninformative prior distributions on all parameters. We assumed diffuse normal priors for the fixed effects *β*, *α*, *γ_δ_*, and *γ_φ_* with a mean of zero and a precision of 0.0001. Following the recommendations of Gelman and Hill [39] we assigned the site effects *δ_j_*, *φ_j_*, *δ_sj_*, *φ_sj_*, *δ_hj_*, and *φ_hj_* normal prior distributions with a mean of zero and a standard deviation that was uniformly distributed from 0 to 20. *Uniform* [0,20] prior distributions on *σ_h_* and *σ_s_* resulted in a roughly “fair” prior expectation of E[*ψ*]≈0.5 and was relatively flat. For *ω*_1_ and *ω*_2_ we assumed uniform prior distributions from 0 to 100, while *θ_φ_* and *θ_δ_* had uniform prior distributions from 0 to 3. We also investigated the effects of the prior distributions by using improper uniform priors across the whole real line (dflat() in WinBUGS parameterization) for fixed effects and uniform priors from 0 to 100 for the standard deviations of the site effects. These changes had little effect on our parameter estimates or the relative ranking of models by the deviance information criterion (DIC).

The DIC statistic, developed by Spiegelhalter *et al.* [40], approximates the popular AIC statistic [41] in the Bayesian context. The DIC was computed as DIC = 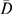
+*pD*, where 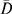 is the posterior mean deviance and *pD* equals 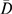 minus the deviance calculated with parameters set to their posterior mean 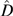. The smaller the DIC value, the better the model [40]. As suggested by Knorr-Held and Richardson [42], we view the DIC values as rough indices for model evaluation, but also used the posterior distributions to assess the importance of model parameters and relative merit of different model structures. Models that include so-called “random” effects may be sufficiently flexible to fit the data while providing few biological insights about why groups or sites differ. Thus, we often prefer the hierarchical models that attempt to explain why sites differ even though they may have similar, or worse, DIC values.

We used the R2WinBUGS package to call WinBUGS version 1.4.3 [43] from R version 2.9 [44]. All models were run for 20,000 iterations on three different Markov chains and the first half of each chain was discarded. Models including elk density as a non-linear effect took longer to converge, from 100,000 to 2 million iterations. We assessed convergence using the Gelman-Rubin-Brooks statistic, where 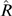
<1.1 for all parameters indicated that relatively little variation was associated with specific MCMC chain [39]. To predict the site-level seroprevalence we added an additional record to the dataset for each hunt area and its covariates with Year equal to 1991 or 2009. We then used WinBUGS to estimate the missing response variable, *p* for site *j*, assuming that all individuals were from management-related captures rather than hunted samples (i.e., Hunt  = 0).

## Results

**Figure 2:**
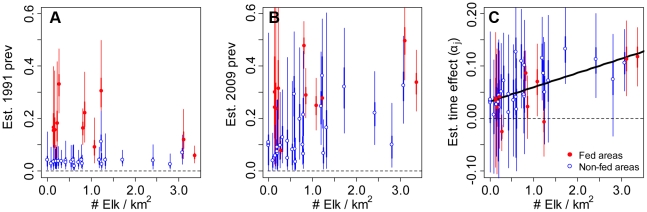
Model-based estimates of brucellosis seroprevalence among adult female elk as a function of elk density at the hunt area scale. The estimates for 1991 (A), 2009 (B) and the temporal trend (C) were based on the means of the predictive posterior distributions for Model 1 and were standardized by assuming all samples were from research captures. In (C), the temporal trend is on the logit scale, whereby <I>{lower case alpha}<sub>j</sub></I> is the change in the log-odds of being test-positive in site <I>j</I> associated with a one-year increase in time. The wide and thin lines refer to the 50 and 95% credibility intervals, respectively. Red solid circles represent regions that contained supplemental elk feeding grounds. Regions without feeding grounds are represented by blue open circles.

We report results in the format of parameter: posterior mean (posterior standard deviation). In our initial set of *a priori* models, models that assumed hunt areas with the same covariates were exchangeable (EX) generally performed better than spatial convolution models that accounted for correlations among neighboring areas (BYM, Table S2). BYM models tended to have higher DIC values compared to similarly structured EX models, and the posterior distributions of *ψ_β_* and *ψ_α_* shifted downwards compared to the prior expectation (*ψ_β_*: 0.40 (0.04) and *ψ_α_*: 0.40 (0.04), Model 12) indicating that the random heterogeneity effects were more important than the spatial neighborhood effects. Models that included site effects on the intercept *δ_j_* and slope *φ_j_* tended to fit the data better, indicating that areas differed in their 1991 seroprevalence as well as their change over time (Table S2). Elk population density was either uncorrelated, or negatively correlated, with the starting seroprevalence in 1991 depending on whether two outlier sites with feeding grounds were included (Fig. 2). Sites with feeding grounds had higher 1991 seroprevalences but there was only weak support for a fed by time interaction (*β*_fed:_ 2.70(0.44) *α*_fed_: −0.06(0.03), Model 8). In our subsequent analyses we removed parameters from the a priori model set whose 95% credible intervals overlapped zero and included the effects of test and remove, hunted elk vs. management captures, and elk population density.

**Figure 3:**
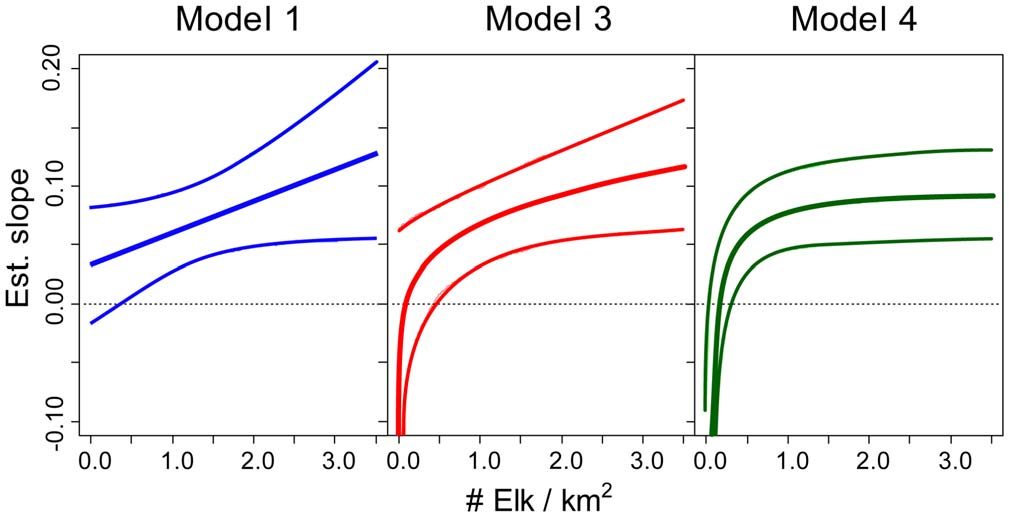
The overall relationship between elk density and the annual rate that brucellosis is increasing on the logit scale for three of the top models. The relationship is constrained to be linear (Model 1), a power function (Model 3) or a saturating type II response (Model 4). Thin lines are the 95% credibility intervals.

When entered in a linear form, there was a 96% probability that population density was positively associated with the temporal increase in seroprevalence (*γ_φ_*: 0.027(0.015), Model 1, Fig. 2). Comparing hunt areas that differ by one elk/km^2^, after 19 years a subject in the higher density hunt area is about one and half times more likely to be test positive, which is the difference between 20 and 30 percent seroprevalence (Fig. 2). When we modeled the density effect as a power function (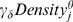, Model 3), the posterior distribution of *θ* overlapped zero (Fig. S2), but the average effect of density was still positive (Figs. 3 and S2; *θ*: 0.23(0.34)). Models without the density effect, or with density modeled as a nonlinear effect, were within two DIC units of the top linear model (Fig. 3, Table 1). Thus, the data did not strongly differentiate between these model structures. There was little difference in the 2009 seroprevalence predictions for top models (Fig. 3, S3, S4).

**Figure 4:**
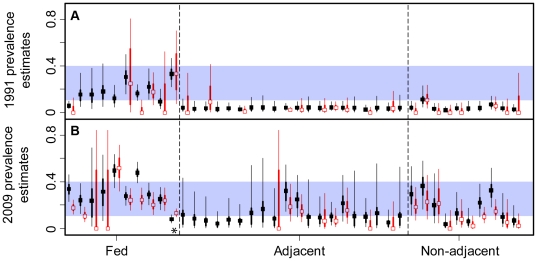
A comparison of model-based and raw data estimates of brucellosis seroprevalence among adult female elk. Model estimates for 1991 (A) and 2009 (B) were based on the means of the predictive posterior distributions from Model 1 (black). Raw estimates were based on data from 1991–1994 (A) and 2006–2009 (B; red, offset to the right). Lines refer to the credibility and confidence intervals for the model and empirical estimates, respectively (wide lines  = [25–75], thin lines  = [2.5–97.5]). Fed areas were regions that included a supplemental elk feeding grounds. Adjacent areas shared a boundary with fed areas. Non-adjacent areas did not share a boundary with areas with feedgrounds. Model estimates were standardized by assuming all samples were from research captures. The asterisk marks the test-and-remove region and the empirical estimate was based only on 2009 data for that site. The blue rectangle highlights the range of seroprevalence estimates of fed regions in 1991, which included some regions without feedgrounds in 2009.

The type of sample (hunter sample or management captures) was a strong predictor of the test results (*β*_hunt_: −0.79 (0.21), Fig. S2, Model 1). Thus, the odds a hunted elk was positive was less than half that of a management capture (*e* ^−0.79^ = 0.45) while controlling for location and year. To further control for any potential confounding we ran Model 1 using only data from the regions with feeding grounds for 1993–2008 and the difference between hunter samples and management captures was even stronger (*β*_hunt_:−0.89 (0.21)). Our model estimates of site-specific seroprevalence assumed that all samples came from management captures as a way of standardizing the sampling regime to facilitate comparison among areas. This is similar to standardizing according to age or sex [45]. As a result, the model estimates in some regions tend to be higher than the crude seroprevalence estimates (Fig. 4), which have a mix of hunter and management samples.

**Figure 5:**
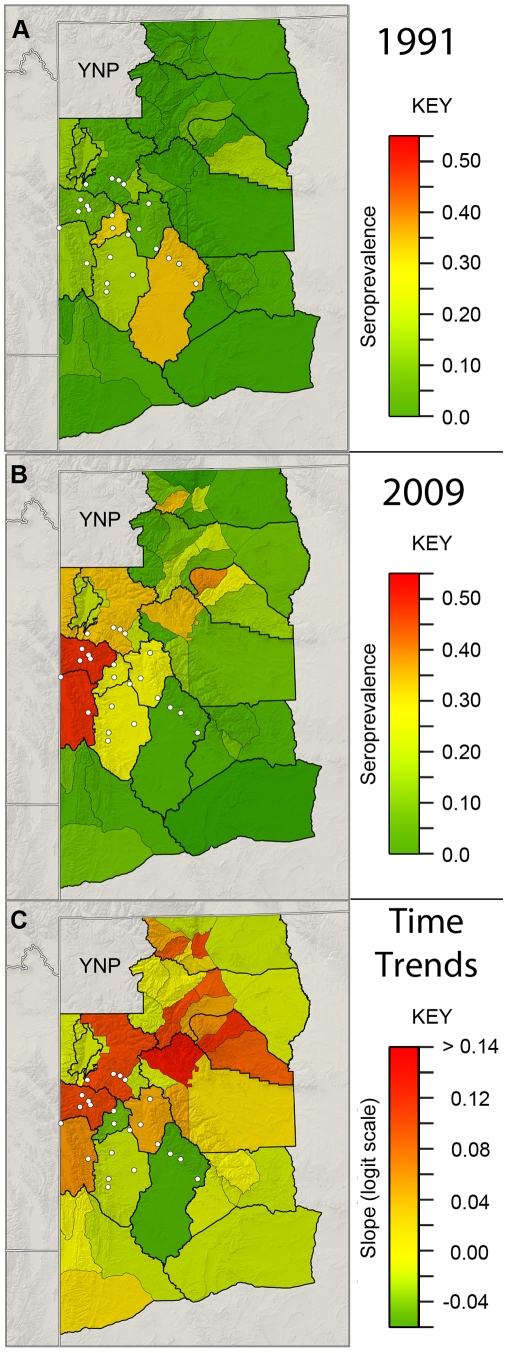
Maps of the brucellosis seroprevalence estimates for adult female elk and the annual trends. The estimates for 1991 (A), 2009 (B) and the temporal trend (C) were based on the means of the predictive posterior distributions for Model 1 and were standardized by assuming all samples were from research captures. The temporal trend is on the logit scale. Supplemental feeding grounds are represented by white circles.

Very few areas outside of the hunt areas with feeding grounds had a seroprevalence of over 3% in 1991, but by 2009 there were several hunt areas non-adjacent to the feeding grounds and east of Yellowstone National Park that had seroprevalence estimates over 20%, similar to feeding ground regions (Figs. 4 and 5A and B). In contrast, areas south of the feeding grounds showed little to no increase in seroprevalence (Fig. 5C). Within the regions with supplemental feeding, one region (HAs 97 & 98) in the south east of the GYE had strong decreases in seroprevalence over time that appeared to be associated with the test and remove program from 2006–2009 (*α*_tr_: −0.44(0.09); Fig. S5). Meanwhile, hunt areas adjacent to Idaho increased in seroprevalence and had the highest seroprevalence in 2009. Models that used data aggregated at the coarser herd unit scale required fewer site-specific parameters, but fit the data less well and had DIC values that were more than 10 units higher than similar models at the hunt area scale (Table 1).

## Discussion

Few datasets exist for natural systems that address the relationship between host density and pathogen transmission [but for timeseries analyses see: 46], [47], [48]. We showed that the seroprevalence of brucellosis in Wyoming elk is increasing in some regions where elk are not artificially aggregated onto supplemental feeding grounds and these increases in seroprevalence are correlated with elk densities at the hunt area scale (Fig. 2 and S2). However, the available data could not differentiate among linear and non-linear effects of host density (Fig. 3, Table 1), which is critical to management efforts. If additional data support a saturating functional response (Model 4, Fig. 3C) then management efforts targeting elk density are unlikely to affect brucellosis dynamics unless elk are reduced to very low densities, an unpopular scenario for sportsperson and conservation groups.

Collecting host and pathogen data at the appropriate spatial scale is critical to estimating the relationship between host density and pathogen transmission. We suspect that issues of spatial scale underlie much of the unexplained variation in this relationship (Fig. 2). Group size distributions of many social species are right skewed, with many small groups and a few large groups [10], [49]. This is also true for elk [19]. Disease dynamics are likely to be driven by these large groups, but the number and size of these large groups may be only weakly correlated with the overall density of the region. As a result, researchers may try to collect data at the group level, but even this may not clarify the relationship. Even when transmission occurs only in the largest groups, movement among groups may obscure the relationship, particularly for serological datasets.

These issues of spatial scale and the relationship between host density and pathogen transmission have strong management implications. Reducing host densities at a regional scale may have little effect on the largest groups. For example, increased hunting quotas may reduce overall elk densities and yet have no effect upon the size of the largest groups if those groups exist in areas with little to no hunting. Temporal scale is also an important consideration. The effects of hunting and wolf predation on elk group sizes may differ depending on the timescale. In the short-term, wolves and hunters may concentrate elk due to behavioral effects, while the longer-term demographic effects may reduce elk aggregations.

Although a few elk populations in the GYE are declining, many populations are growing in Montana, Wyoming, and nationwide [19], [50], [51]. While average elk group sizes in the GYE are relatively constant, the largest groups are getting larger as elk populations increase [19]. As a result, elk may now be maintenance hosts for brucellosis in new regions of the GYE, which is likely to complicate U.S. Department of Agriculture eradication efforts. Areas outside the GYE with large elk populations may support brucellosis in the future if *B. abortus* is introduced.

Not surprisingly, the hunt areas with the highest seroprevalence in 1991 were those that contained supplemental feeding grounds. By 2009, however, several regions distant from the feeding grounds had increased in seroprevalence to levels comparable to feeding grounds (Figs. 2, 4, and 5). Hunt areas 97 and 98 showed a strong decrease in seroprevalence from 2006 to 2009 that is coincident with a WGFD test-and-remove program of seropositive elk on three supplemental feeding grounds (Fig. 5C and S5). Although the test-and-remove program appears to reduce seroprevalence, whether or not this reduction is worth the economic costs is the subject of ongoing discussion and research.

Hunter-killed elk were less likely to be test positive than management captures after accounting for location and year. With an estimated odds ratio of 0.45, we would expect that a region with a seroprevalence of 15% based upon management captures would have a seroprevalence of only 7.5% from hunter samples. We postulate that this effect may be due to four different mechanisms. First, hunter samples may be of lower quality than management-related captures perhaps due to inadequate refrigeration or delays between killing the animal and collecting the blood sample. Second, the feedground captures occur in January-March while hunting typically occurs in September-December; and elk may be more likely to be test-positive as their pregnancies progress. Third, hunters may be sampling a different population of individuals than those that are captured on the feeding grounds. Finally, captures on feeding grounds may be more likely to test positive due to higher levels of other pathogens on the feeding grounds, such as *Yersinia enterocolitica*, that may cross-react with the brucellosis serological tests [52], [53].

In many of the hunt areas with feeding grounds, over 80% of elk were located on feeding grounds during the winter [54]. Therefore, the differences in the probability of being seropositive between hunter samples and feedground captures was probably not entirely due to hunter samples having a higher proportion of non-feedground elk in regions with feeding grounds. In addition, the WGFD only tests those hunter samples that have not undergone a significant amount of red blood cell lyses, so we believed the quality was acceptable for antibody tests. The relative effects of cross-reactions and testing during later stages of pregnancy remain unknown. If cross-reactions were responsible then captures on feeding grounds may overestimate brucellosis seroprevalence. If females were more likely to test positive later in pregnancy, then hunter samples may underestimate seroprevalence relative to management captures. More work is necessary to differentiate these possibilities.

This study is the most refined analysis of host density effects for brucellosis to date; however, there are several avenues for future research. Our population density estimates assume that the entire hunt area is suitable elk habitat. This could be further refined by excluding areas of unsuitable habitat and collecting data on group size distributions in different regions. Second, our previous work on the feeding grounds suggests that understanding how elk densities vary during the transmission period of February to June is critical [15], [27]. The likelihood of abortion for infected individuals varies over time; as a result, high densities for short periods may have equivalent transmission rates to areas with lower densities that are present for the entire transmission period. Finally, we related spatial differences in elk density to the spatio-temporal changes in brucellosis seroprevalence. A more complete analysis would account for how elk densities have changed over time as well as space. This analysis, however, would be complicated by the long time lags inherent in serology data of a long-lived host and, unlike more acute infections, temporal changes in brucellosis seroprevalence are relatively slow (Fig. S5).

## Supporting Information

Table S1 Characteristics of the Wyoming hunt areas and herd units used in the analysis.
(0.10 MB DOC)

Table S2 Comparison of a priori models using hierarchical Bayesian logistic regressions of 6458 brucellosis test results of adult female Wyoming elk.
(0.04 MB DOC)

Figure S1 Map of the most recent elk density estimates from 2004 to 2007. Elk densities were based upon aerial trend counts divided by the area of the unit. Sites labeled with an asterisk did not have any trend count data.
(2.70 MB TIF)

Figure S2 Kernel density estimates of the posterior distributions for four parameters in Model 1 (black), and Model 3 (red). *β*_hunt_ represents the difference between hunter samples and management captures (A). *β*_fed_ represents the increased 1991 seroprevalence associated with supplemental feeding grounds (B). *γ* and *θ* defined the relationship between elk density and the increases in brucellosis over time (*γ*Density*^θ^*; C, D, and E). Note that the scales change among plots.
(0.53 MB TIF)

Figure S3 Comparison of model estimates of elk brucellosis prevalence in 1991 (lower half) and 2009 (upper half) using models 1, 2, 3, 7 and 8 (Table 1). The dashed line is a 45 degree line representing an exact correspondence among model estimates.
(0.31 MB TIF)

Figure S4 Means of the predictive posterior distributions for Models 1, 2 and 3 (columns from left to right; Table 1) of the 1991 prevalence (row 1), 2009 prevalence (row 2), and the annual time trend (row 3) measured on the logit scale. All seroprevalence estimates were standardized by assuming samples were from management captures.
(7.08 MB TIF)

Figure S5 Timeseries of brucellosis seroprevalence in all the hunt areas of northwestern Wyoming that had positive tests. Red squares and lines represent the raw estimates and 95% confidence intervals calculated directly from the empirical data on an annual basis. Black lines represent the mean of the predictive posterior distributions based on Model 1 for each hunt area assuming that all samples were research captures. Areas with supplemental feedgrounds are in the top two rows. Hunt areas 97 and 98 included a test-and-remove effect for 2006-2009.
(0.74 MB TIF)
