# Supplementary material for: Mapping Brucellosis Increases Relative to Elk Density Using Hierarchical Bayesian Models
Source: PLoS One. 2010 Apr 23;5(4):e10322. doi: 10.1371/journal.pone.0010322 (PMC2859058; doi:10.1371/journal.pone.0010322)
Supplement: Table S1 — Characteristics of the Wyoming hunt areas and herd units used in the analysis. (0.10 MB DOC) [file pone.0010322.s001.doc]

Table S1. Characteristics of the Wyoming hunt areas and herd units used in the analysis.

| Hunt areas | Herd unit | Order^1^ | Fed^2^ | Disease tests | # positive | Area (km^2^) | # Elk/km^2^ |
| --- | --- | --- | --- | --- | --- | --- | --- |
| 73 | 101 | 1 | 1 | 1 | 0 | 1082 | 0.138 |
| 70, 71, 77, 80-82 | 102 | 2 | 1 | 552 | 73 | 3557 | 3.364 |
| 72, 74, 75, 79 | 102 | 3 | 1 | 153 | 17 | 1288 | 0.134 |
| 83 | 102 | 4 | 1 | 2 | 0 | 429 | 0.163 |
| 78 | 102 | 5 | 1 | 12 | 2 | 95 | 0.220 |
| 84, 85 | 103 | 6 | 1 | 113 | 34 | 1767 | 3.104 |
| 86, 87 | 104 | 7 | 1 | 223 | 64 | 767 | 1.225 |
| 88-91 | 105 | 8 | 1 | 434 | 141 | 2511 | 0.809 |
| 92, 94 | 106 | 9 | 1 | 232 | 53 | 4082 | 0.850 |
| 93, 95, 96 | 107 | 10 | 1 | 126 | 24 | 2147 | 1.077 |
| 97, 98 | 108 | 11 | 1 | 939 | 194 | 6306 | 0.272 |
| 103 | 428 | 12 | 0 | 35 | 1 | 684 | 0.423 |
| 104 | 428 | 13 | 0 | 15 | 0 | 1749 | 0.596 |
| 102 | 428 | 14 | 0 | 66 | 1 | 4014 | 0.207 |
| 100 | 426 | 15 | 0 | 37 | 0 | 10237 | 0.091 |
| 99 | 637 | 16 | 0 | 98 | 2 | 1783 | 0.186 |
| 28 | 637 | 17 | 0 | 230 | 3 | 1408 | 0.789 |
| 127 | 635 | 18 | 0 | 0 | 0 | 7990 | NA |
| 69 | 635 | 19 | 0 | 0 | 0 | 861 | 1.324 |
| 68 | 635 | 20 | 0 | 12 | 0 | 555 | 0.542 |
| 67 | 635 | 21 | 0 | 806 | 33 | 1812 | 1.718 |
| 59 | 216 | 22 | 0 | 198 | 10 | 1017 | 1.192 |
| 60 | 216 | 23 | 0 | 1 | 0 | 855 | 0.000 |
| 55 | 216 | 24 | 0 | 123 | 3 | 1011 | 0.196 |
| 56 | 216 | 25 | 0 | 347 | 10 | 1237 | 0.776 |
| 58 | 216 | 26 | 0 | 108 | 5 | 637 | 0.788 |
| 66 | 216 | 27 | 0 | 0 | 0 | 4958 | 0.000 |
| 61 | 216 | 28 | 0 | 202 | 3 | 1021 | 2.804 |
| 105 | 428 | 29 | 0 | 0 | 0 | 4399 | NA |
| 25 | 637 | 30 | 0 | 122 | 2 | 2653 | 0.425 |
| 27 | 637 | 31 | 0 | 0 | 0 | 306 | 0.000 |
| 63 | 214 | 32 | 0 | 142 | 9 | 948 | 0.586 |
| 62 | 214 | 33 | 0 | 135 | 16 | 662 | 1.221 |
| 64 | 214 | 34 | 0 | 100 | 3 | 2656 | 0.714 |
| 50 | 217 | 35 | 0 | 135 | 0 | 925 | 0.608 |
| 51 | 217 | 36 | 0 | 175 | 5 | 675 | 0.318 |
| 65 | 217 | 37 | 0 | 50 | 1 | 4351 | 0.146 |
| 54 | 217 | 38 | 0 | 95 | 9 | 349 | 2.415 |
| 52 | 217 | 39 | 0 | 268 | 23 | 448 | 3.074 |
| 53 | 217 | 40 | 0 | 84 | 2 | 159 | 0.258 |
| 121 | 217 | 41 | 0 | 87 | 1 | 471 | 1.247 |

1. Order of appearance from left to right in Figure 4.

2. Whether or not an area includes a supplemental feeding ground.

3. Number of elk from the most trend count since 2004. (Wyoming Game and Fish Department (2007) Annual big game herd unit job completion reports. Cheyenne, Wyoming.)
