# Supplementary material for: Mapping Brucellosis Increases Relative to Elk Density Using Hierarchical Bayesian Models
Source: PLoS One. 2010 Apr 23;5(4):e10322. doi: 10.1371/journal.pone.0010322 (PMC2859058; doi:10.1371/journal.pone.0010322)
Supplement: Table S2 — Comparison of a priori models using hierarchical Bayesian logistic regressions of 6458 brucellosis test results of adult female Wyoming elk. (0.04 MB DOC) [file pone.0010322.s002.doc]

Table S2. Comparison of *a priori* models using hierarchical Bayesian logistic regressions of 6458 brucellosis test results of adult female Wyoming elk.

| Model | Intercept covariates*^1^* | Time covariates | Intercept*^2^* | Time | DIC*^3^* | ΔDIC | pD*^4^* | Deviance |
| --- | --- | --- | --- | --- | --- | --- | --- | --- |
| 8 | HA, Fed | HA, Fed | EX | EX | 3936.3 | 39 | 33.9 | 3909.0 |
| 9 | HA | HA | EX | EX | 3937.1 | 39 | 40.8 | 3897.0 |
| 10 | HA, Pop | HA, Pop | EX | EX | 3937.8 | 40 | 40.0 | 3897.8 |
| 11 | HA, Fed, Pop | HA, Pop, Fed | EX | EX | 3937.9 | 40 | 33.5 | 3904.4 |
| 12 | HA, Fed | HA, Fed | BYM | BYM | 3941.7 | 44 | 36.7 | 3905.0 |
| 13 | HA, Fed, Pop | HA, Pop, Fed | BYM | BYM | 3942.7 | 45 | 36.5 | 3906.2 |
| 14 | HA | HA | BYM | BYM | 3946.5 | 49 | 45.6 | 3900.9 |
| 15 | Fed | HA, Fed, Pop | none | EX | 3977.6 | 80 | 22.6 | 3955.1 |
| 16 | HA, Fed | Fed | EX | None | 3978.1 | 80 | 22.9 | 3955.2 |
| 17 | Fed | HA, Fed | none | EX | 3979.7 | 82 | 22.7 | 3957.0 |
| 18 | HA, Fed | Fed | BYM | None | 3980.8 | 83 | 24.1 | 3962.0 |
| 19 | Fed | HA, Fed | none | BYM | 3983.5 | 86 | 24.9 | 3964.0 |
| 20 | HA | constant | EX | None | 3999.8 | 102 | 27.5 | 3972.2 |
| 21 | HA | constant | BYM | None | 4006.8 | 109 | 30.7 | 3981.0 |
| 22 | constant | HA | none | EX | 4095.2 | 198 | 27.2 | 4068.0 |

*^1^* Dependent variables that affected the intercept (i.e. 1991 seroprevalence) and the slope (i.e. time effect). HA = Hunt area; Fed = 1 for areas with a supplemental feedground, otherwise 0; Pop = elk/km^2^.

*^2^*Regional intercepts and slopes were either treated as exchangeable (EX) or ere constrained by neighbors that shared a border according to a Besag-York-Mollie (1991; BYM) spatial convolution model.

*^3^* Deviance information criterion

*^4^* pD = Dbar - Dhat, an approximation of the model complexity.
