# Supplementary figures and images for: Mapping Brucellosis Increases Relative to Elk Density Using Hierarchical Bayesian Models
Source: PLoS One. 2010 Apr 23;5(4):e10322. doi: 10.1371/journal.pone.0010322 (PMC2859058; doi:10.1371/journal.pone.0010322)

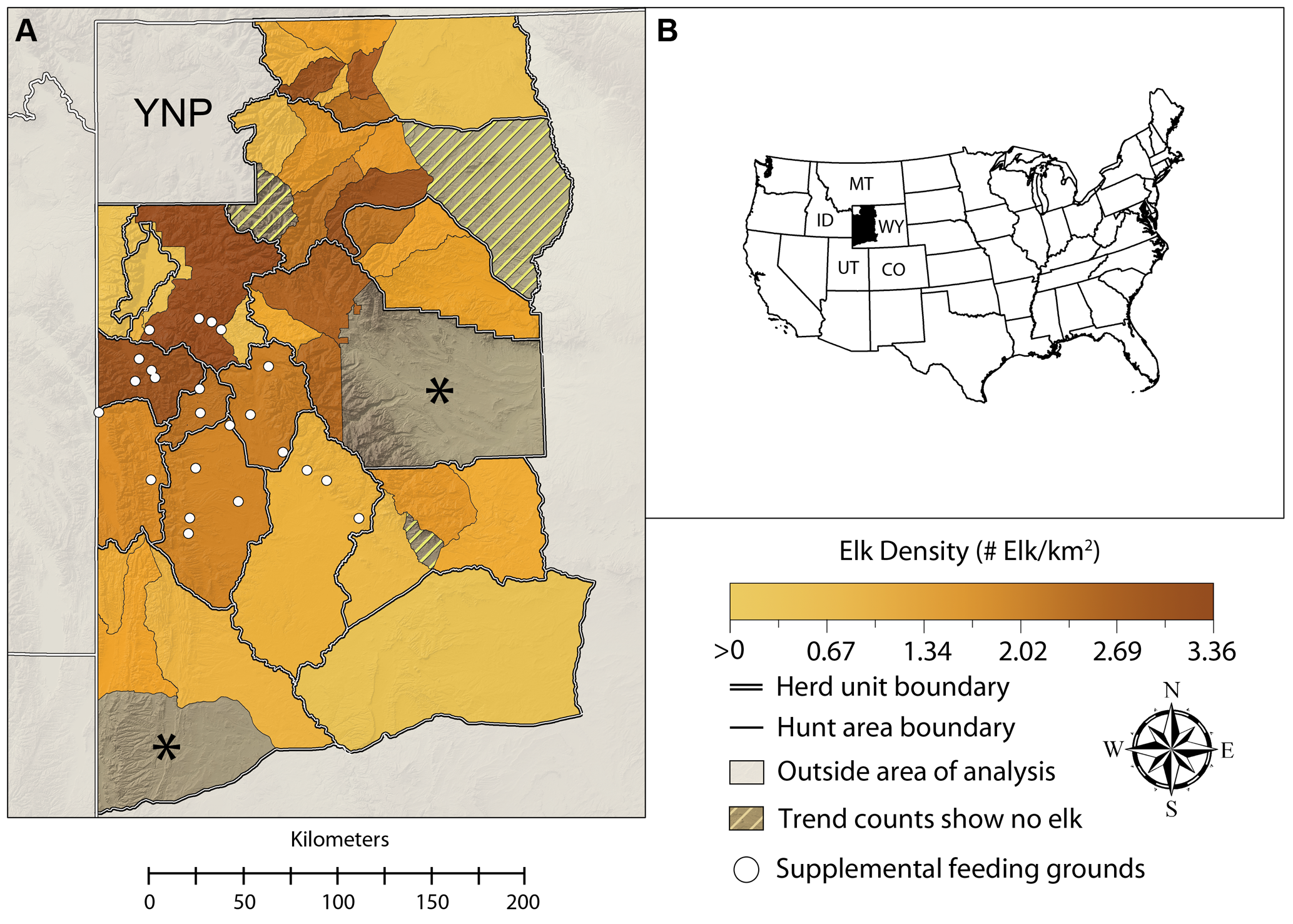

Supplement: Figure S1 — Map of the most recent elk density estimates from 2004 to 2007. Elk densities were based upon aerial trend counts divided by the area of the unit. Sites labeled with an asterisk did not have any trend count data. (2.70 MB TIF) [file pone.0010322.s003.tif]

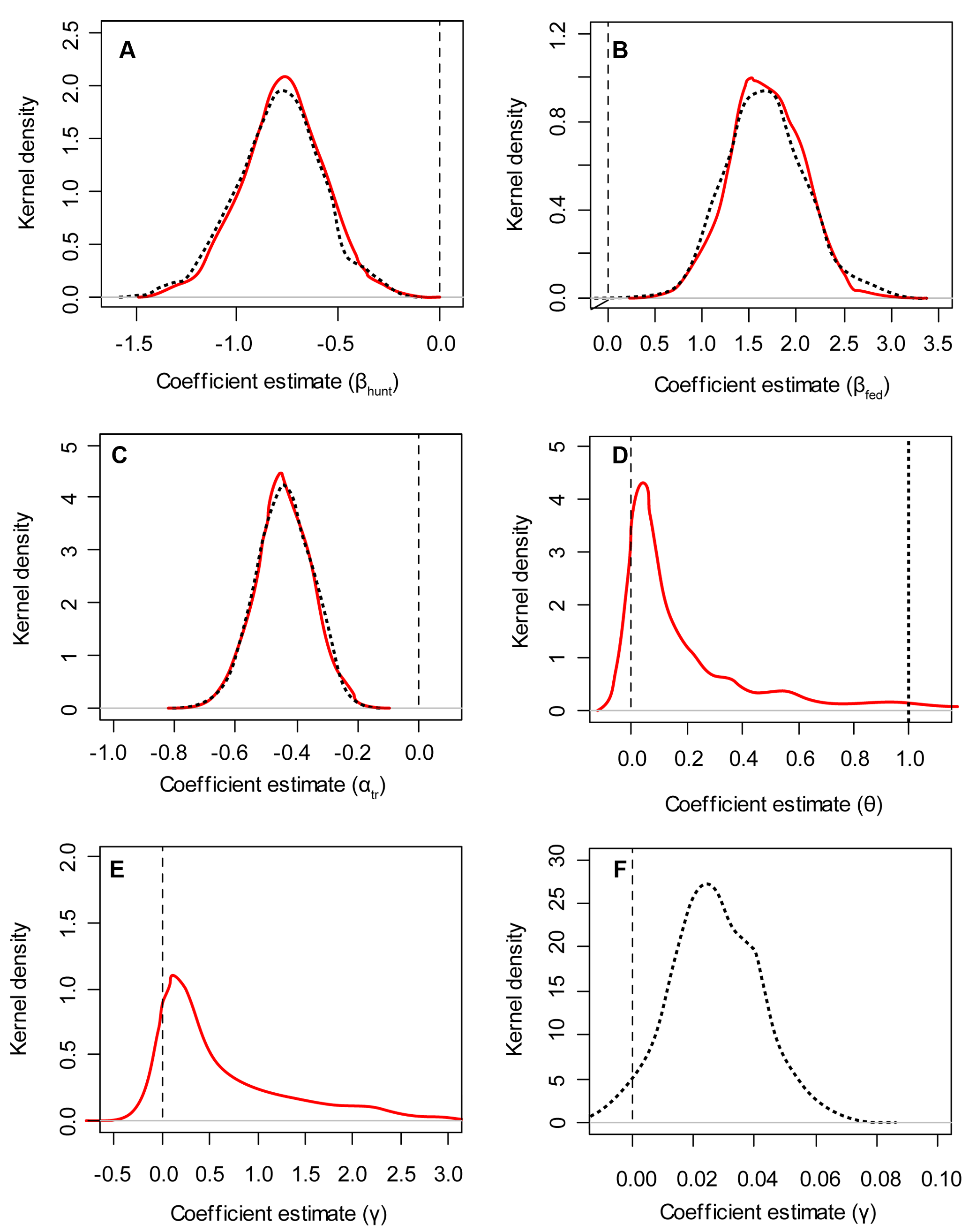

Supplement: Figure S2 — Kernel density estimates of the posterior distributions for four parameters in Model 1 (black), and Model 3 (red). β hunt represents the difference between hunter samples and management captures (A). β fed represents the increased 1991 seroprevalence associated with supplemental feeding grounds (B). γ and θ defined the relationship between elk density and the increases in brucellosis over time (γDensityθ; C, D, and E). Note that the scales change among plots. (0.53 MB TIF) [file pone.0010322.s004.tif]

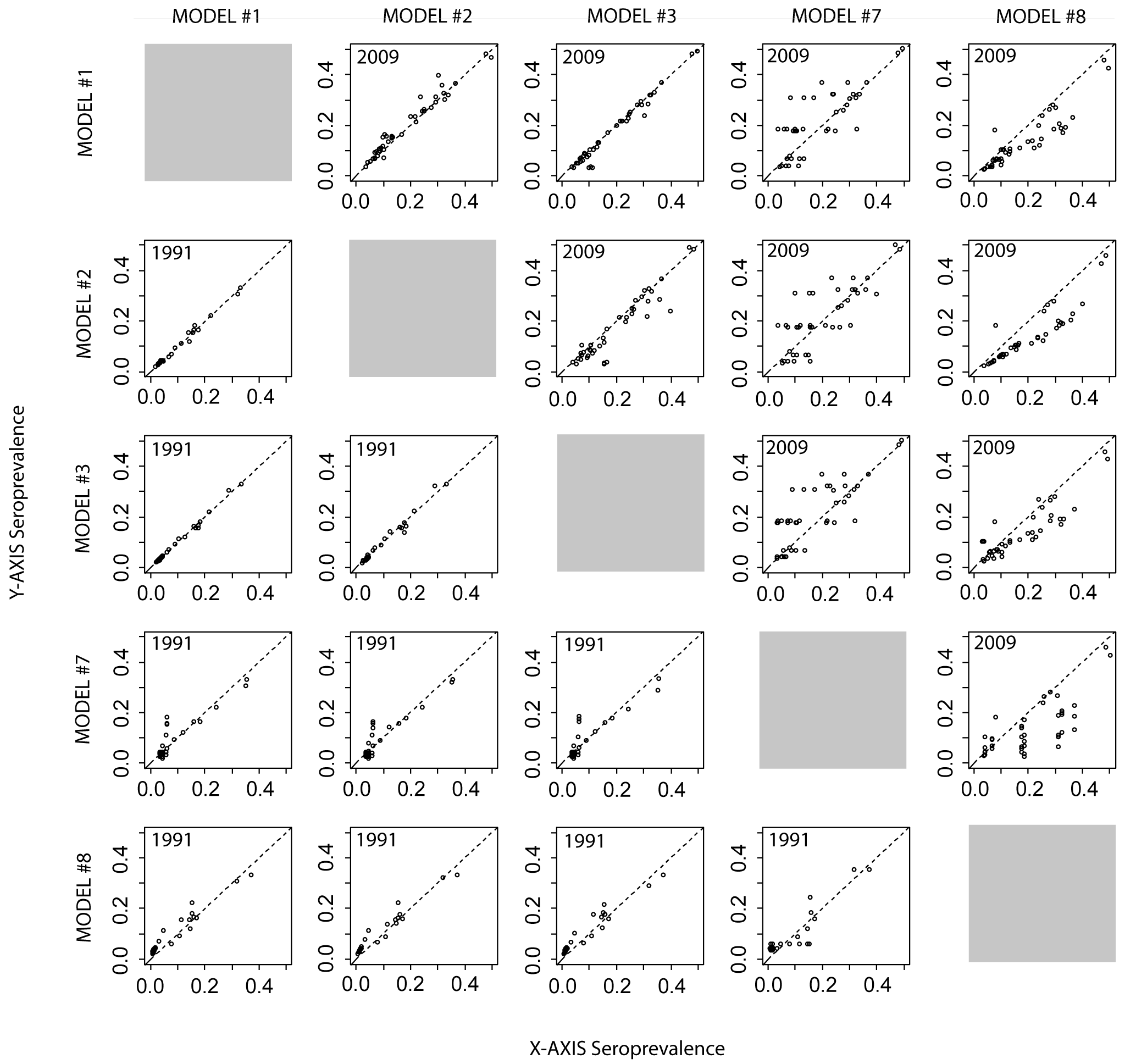

Supplement: Figure S3 — Comparison of model estimates of elk brucellosis prevalence in 1991 (lower half) and 2009 (upper half) using models 1, 2, 3, 7 and 8 (Table 1). The dashed line is a 45 degree line representing an exact correspondence among model estimates. (0.31 MB TIF) [file pone.0010322.s005.tif]

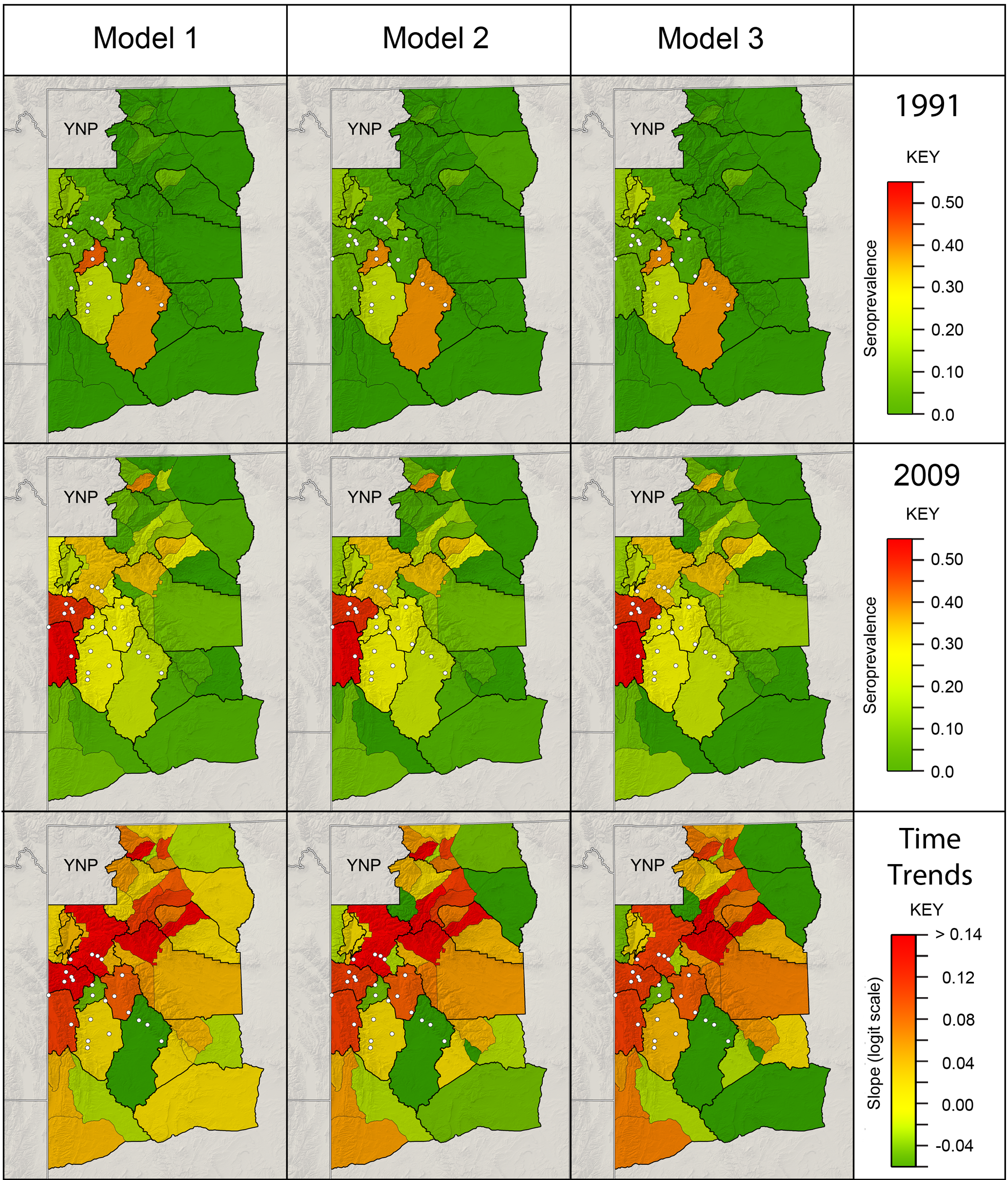

Supplement: Figure S4 — Means of the predictive posterior distributions for Models 1, 2 and 3 (columns from left to right; Table 1) of the 1991 prevalence (row 1), 2009 prevalence (row 2), and the annual time trend (row 3) measured on the logit scale. All seroprevalence estimates were standardized by assuming samples were from management captures. (7.08 MB TIF) [file pone.0010322.s006.tif]

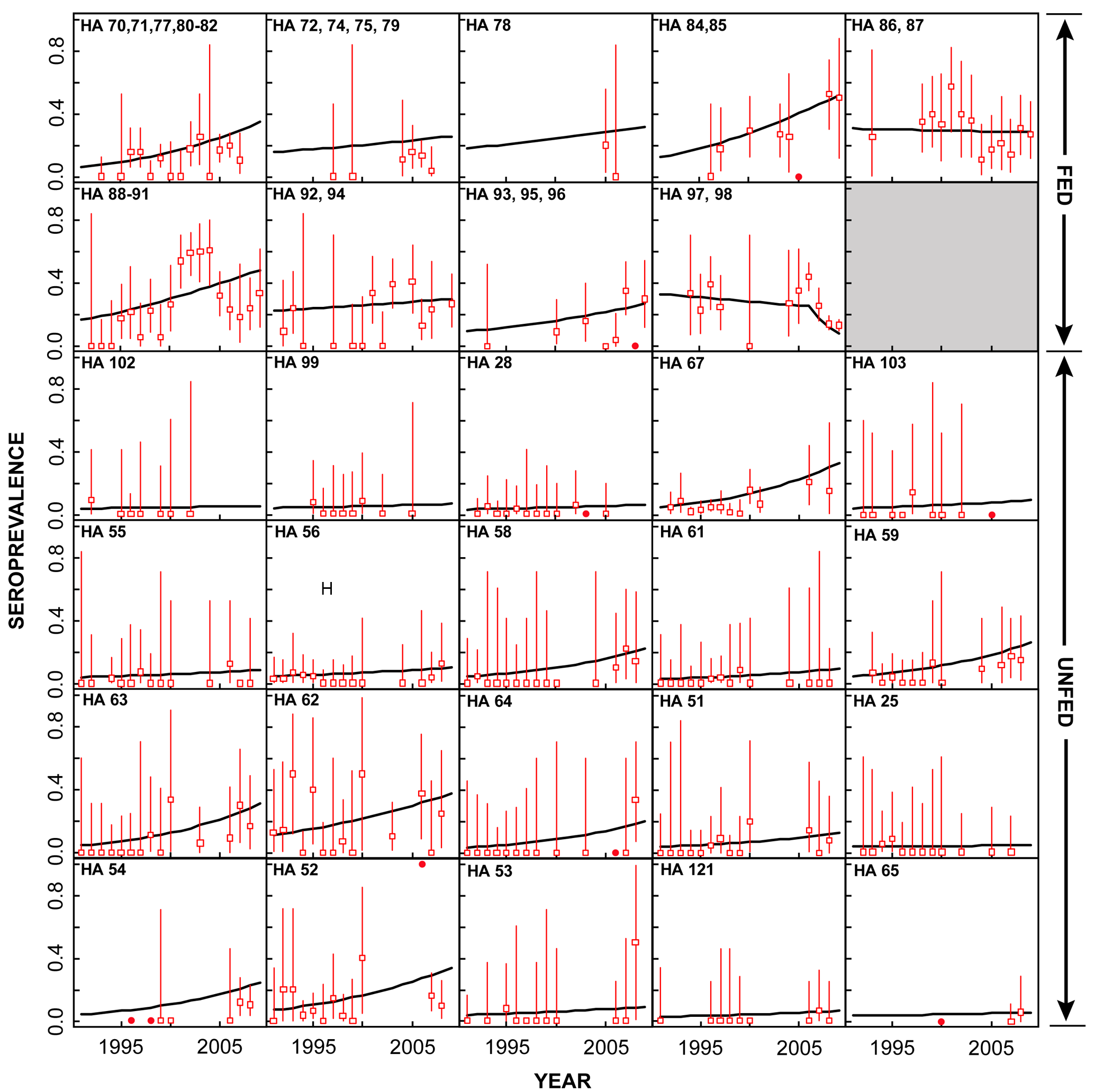

Supplement: Figure S5 — Timeseries of brucellosis seroprevalence in all the hunt areas of northwestern Wyoming that had positive tests. Red squares and lines represent the raw estimates and 95% confidence intervals calculated directly from the empirical data on an annual basis. Black lines represent the mean of the predictive posterior distributions based on Model 1 for each hunt area assuming that all samples were research captures. Areas with supplemental feedgrounds are in the top two rows. Hunt areas 97 and 98 included a test-and-remove effect for 2006-2009. (0.74 MB TIF) [file pone.0010322.s007.tif]
